# Advanced MR Imaging of Gliomas: An Update

**DOI:** 10.1155/2013/970586

**Published:** 2013-06-04

**Authors:** Hung-Wen Kao, Shih-Wei Chiang, Hsiao-Wen Chung, Fong Y. Tsai, Cheng-Yu Chen

**Affiliations:** ^1^Department of Biomedical Imaging and Radiological Sciences, National Yang-Ming University, No. 155, Sec. 2, Linong Street, Taipei 112, Taiwan; ^2^Department of Radiology, Tri-Service General Hospital, National Defense Medical Center, No. 325, Sec. 2, Cheng-Kong Road, Neihu, Taipei 114, Taiwan; ^3^Department of Electrical Engineering, National Taiwan University, No. 1, Sec. 4, Roosevelt Road, Taipei 106, Taiwan; ^4^Imaging Research Center, Taipei Medical University, No. 250, Wu-Hsing Street, Taipei 110, Taiwan; ^5^Department of Radiological Sciences, University of California at Irvine Medical Center, Irvine, 101 The City Drive South, Orange, CA 92868, USA

## Abstract

Recent advances in the treatment of cerebral gliomas have increased the demands on noninvasive neuroimaging for the diagnosis, therapeutic planning, tumor monitoring, and patient outcome prediction. In the meantime, improved magnetic resonance (MR) imaging techniques have shown much potentials in evaluating the key pathological features of the gliomas, including cellularity, invasiveness, mitotic activity, angiogenesis, and necrosis, hence, further shedding light on glioma grading before treatment. In this paper, an update of advanced MR imaging techniques is reviewed, and their potential roles as biomarkers of tumor grading are discussed.

## 1. Introduction

Cerebral gliomas are the most common and devastating primary brain tumors. Although these tumors are traditionally considered to be arising from normal glial cells, the origin of the tumors remains undetermined. More recently, neural stem cells or progenitors are proposed to be the source of glioma [1]. The World Health Organization (WHO) published a classification system of central nervous system tumors in 1979 and subsequently revised the system in 2000 and 2007. In 2007 system, the major neuroepithelial tumors include astrocytic, oligodendroglial, oligoastrocytic, ependymal, and choroid plexus tumors. The grading of gliomas mainly relies on histological features, including cellularity, nuclear atypia, mitotic activity, vascularity, and necrosis, observed on light microscopy with the aid of immunohistochemistry. 

Among the gliomas, astrocytic tumors are the most common and usually divided into circumscribed and diffuse tumors. The circumscribed tumors are generally in lower grade occurring in young patients while the diffuse tumors are the most common cerebral tumors in adults belonging to WHO grades, II, III, and IV [2]. As the names imply, circumscribed tumors, such as pilocytic astrocytoma (WHO grade I), are localized with distinct margin and diffuse tumors are notorious in their propensity to infiltrate surrounding parenchyma, irrespective of the grades. The WHO grade II astrocytomas consist of diffusely infiltrative and well-differentiated fibrillary, protoplasmic, or gemistocytic astrocytes with increased cellularity and nuclear atypia but without mitoses, endothelial proliferation, or necrosis. The WHO grade III astrocytomas, anaplastic astrocytomas, show higher cellularity and nuclear atypia than the WHO grade II tumors with mitoses but without endothelial proliferation or necrosis. The WHO grade IV astrocytomas, glioblastoma (formerly, glioblastoma multiforme), are the most common form of astrocytic tumors occurring in the subcortical white matter of the cerebral hemispheres. Glioblastomas are densely cellular and pleomorphic tumors with highly mitotic activity, endothelial proliferation, and necrosis. While the majority of glioblastomas are primary (>90%), arising de novo with a short clinical history and without a precursor tumor, secondary glioblastoma (<10%) may transform from a lower grade astrocytoma over a period of years [3]. Nonetheless, the histopathological appearances of the primary and secondary glioblastomas are identical. 

More recently, the advance of genetics and molecular knowledge of gliomas have shown exciting values not only in improving the correlation between the diagnosis and prognosis but also in guiding novel therapy of these devastating diseases [1, 4–6]. For example, mutations of the gene encoding isocitrate dehydrogenase 1 (IDH1) are very common in low-grade astrocytomas, anaplastic astrocytomas, oligodendrogliomas, anaplastic oligodendrogliomas, and secondary glioblastomas but very rare in de novo glioblastoma [7, 8]. The fact that similar genetic aberrations exist in a variety of gliomas suggests common progenitor cells of these tumors. In addition to mutations of IDH1, low-grade astrocytomas usually have TP53 mutation while oligodendrogliomas typically show 1p/19q loss [8]. The concurrent deletion of chromosomes 1p and 19q, a result of an unbalanced translocation, is associated with increased chemosensitivity and a better prognosis [9, 10]. The most common genetic alteration in de novo glioblastomas is loss of heterozygosity (LOH) on chromosome 10 [11, 12]. LOH 1p is rare in both de novo and secondary glioblastomas but has been found to correlate with longer survival [13, 14].

Overall, the prognosis of high-grade gliomas remains poor despite advances in diagnosis and therapy. The median survival is 12 to 15 months in patients with glioblastomas and 2 to 3 years in patients with anaplastic gliomas [15, 16]. The treatment failure is thought to stem from complex biology and heterogeneity of the gliomas. Advances of the techniques in neuroimaging have improved the characterization of the physiology and metabolism of the tumors noninvasively, leading to improved diagnosis and better detection of recurrence, as well as improving image-guided biopsy and therapy [17–21]. This paper provides an update of the functional MR imaging of gliomas, with focus on the imaging biomarkers of the pathological stigmas of gliomas, including cellularity, invasiveness, mitotic activity, angiogenesis, and necrosis. 

## 2. MR Imaging of Cellularity and Invasiveness

### 2.1. Tumor Cellularity by Diffusion-Weighted Imaging

**Figure 1:**
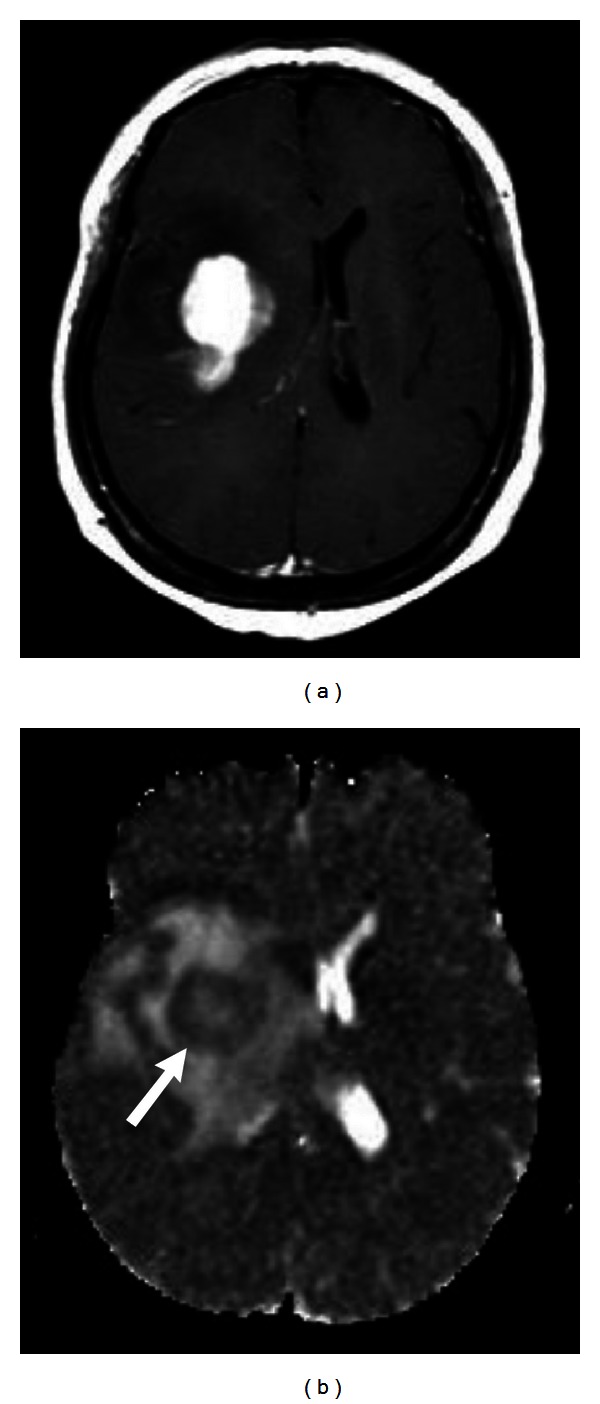
(a) Contrast-enhanced T1-weighted image shows a glioblastoma with strong enhancement after intravenous gadolinium injection. (b) The tumor shows decreased ADC values on ADC map (arrow in (b)).

The cellularity of gliomas can be evaluated by either T2-weighted MR images or diffusion-weighted imaging (DWI) which measures free water molecular diffusion and has been widely used in the diagnosis of acute cerebral infarction and in differentiating tumor necrosis from abscess cavity [22, 23]. In tumor studies, DWI may serve as an early surrogate marker of therapeutic efficacy by implying persistent cellular density in the tumors where high cellularity may impede free water diffusion, resulting in a reduction of apparent diffusion coefficient (ADC) values. Generally, lower ADC values correspond to increased cellularity and high-grade gliomas (Figure 1). This correlation is, however, not linear. In a study by Higano et al. the minimum ADC varies significantly between WHO grade III ((1.06 ± 0.21) × 10^−3^ mm^2^/sec) and WHO grade IV gliomas ((0.83 ± 0.14) × 10^−3^ mm^2^/sec) at *b* value of 1000 sec/mm^2^ [24]. Because the nests of tumor cells tend to be heterogeneous in distribution within the tumor, a measurement of ADC values by manual drawing of the region of interest from the imaging (ADC map) may cause significant sampling bias. A recent study using minimum histogram analysis of apparent diffusion coefficient (ADC) values, instead of mean value, has shown promising correlation with glioma grading [25, 26]. This study using high *b* value and cumulative ADC histogram analysis revealed a significantly higher frequency of low ADC values in high-grade gliomas than those in low-grade ones [26]. The improved analysis methods indeed enhance the role of DWI as a biomarker of tumor cellularity for the diagnosis and monitoring treatment response.

### 2.2. Tumor Invasiveness by Diffusion Tensor Imaging

**Figure 2:**
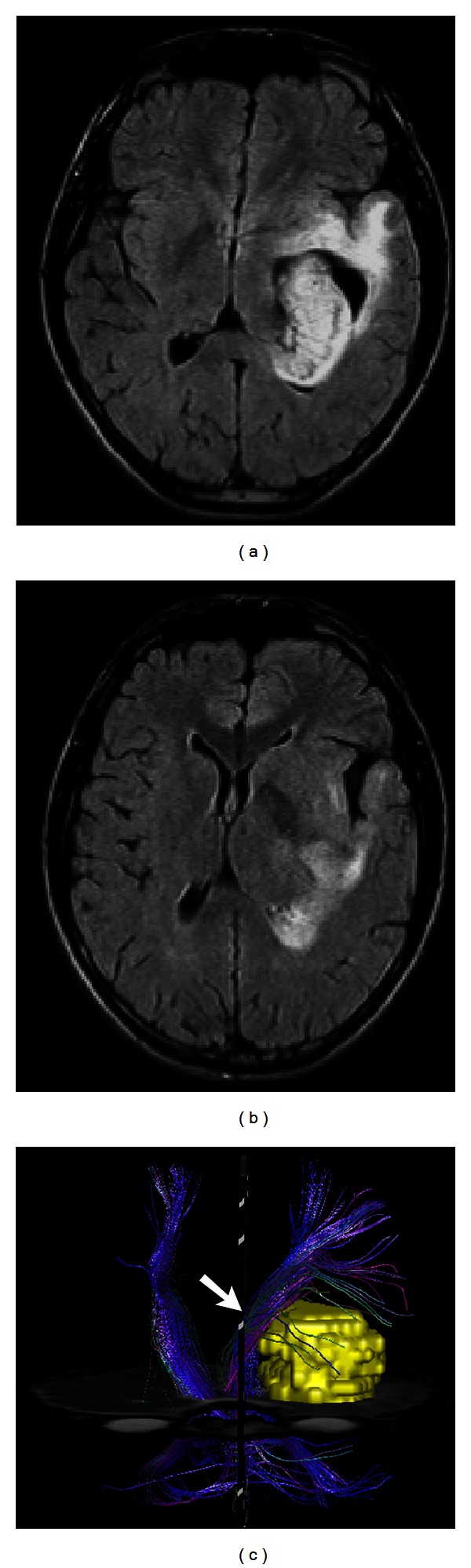
((a), (b)) FLAIR images depict a diffuse and infiltrative oligodendroglioma, WHO grade III, which deviates the corticospinal tract (arrow in (c)) medially, as demonstrated on DTI tractography (c).

**Figure 3:**
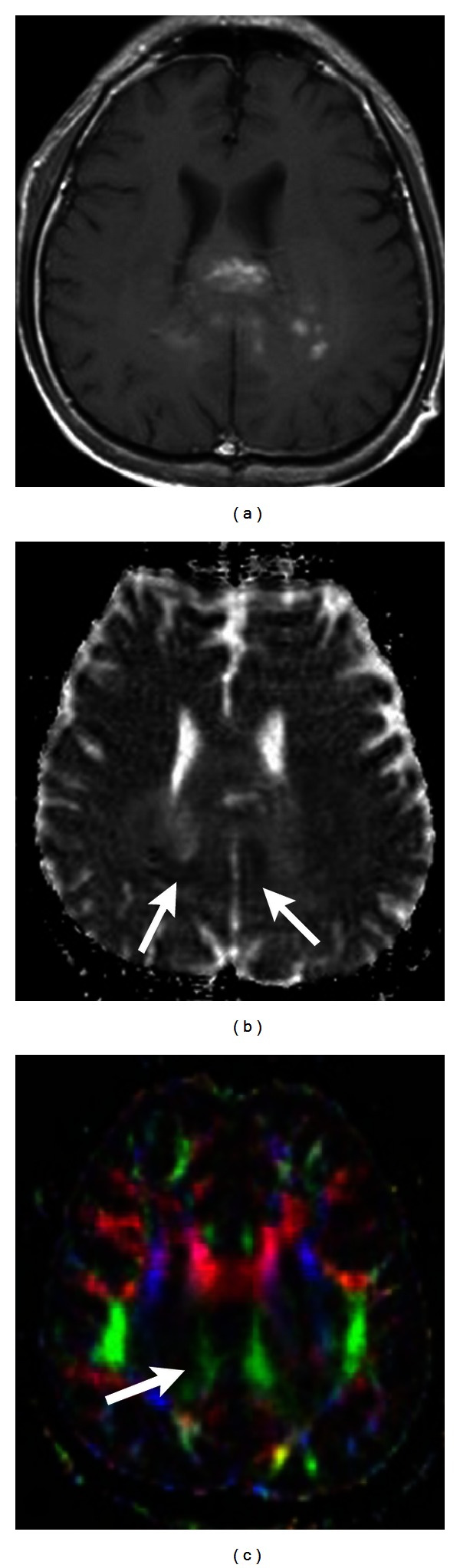
(a) Contrast-enhanced T1-weighted image demonstrates a butterfly glioblastoma involving the genu of corpus callosum with small areas of low ADC value on ADC map (arrows in (b)). On color-coded diffusion tensor imaging, the normal left-right-oriented red color (arrow in (c)) is lost due to destruction of the transverse tracts.

**Figure 4:**
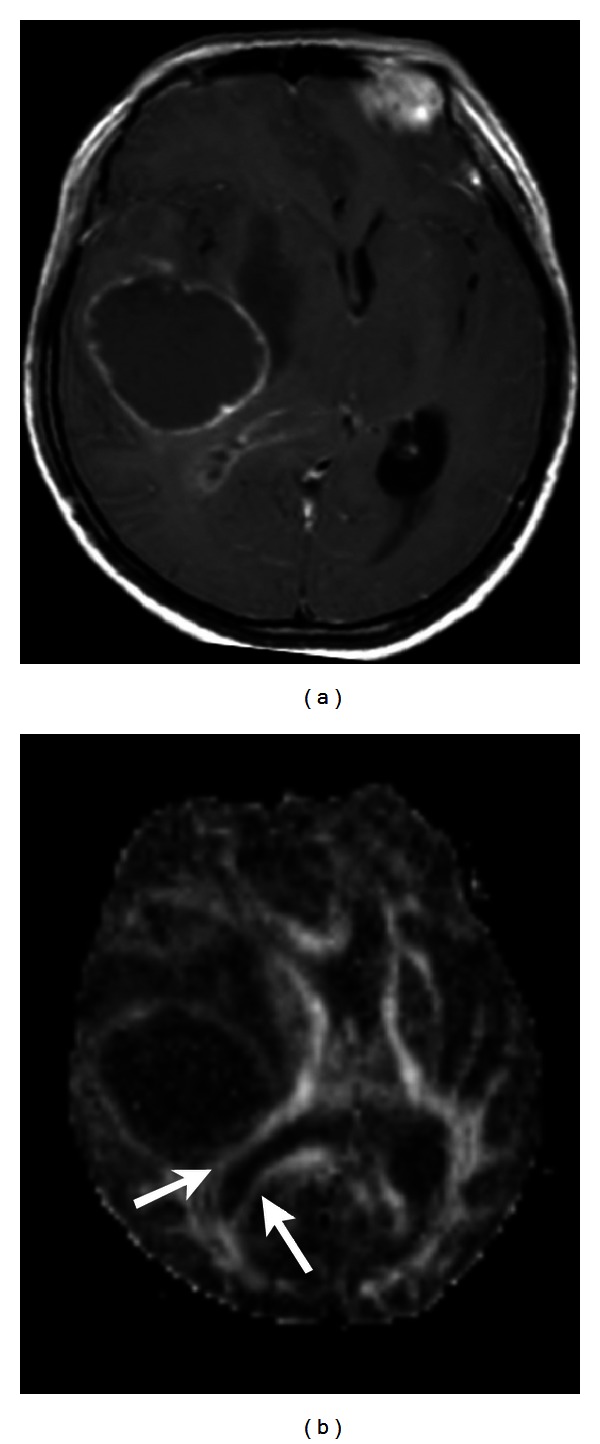
(a) Contrast-enhanced T1-weighted image shows a necrotic glioblastoma with rim-like enhancement. (b) On FA map, attenuated FA (arrows) of the adjacent tracts is shown, indicating tumor infiltration.

Peritumoral invasion is another index of tumor aggressiveness [24]. However, conventional MR imaging cannot accurately evaluate this invasive behavior due to overlapping of the edema and tumor cells. Recent studies have shown a potential role of diffusion tensor imaging (DTI) in this regard [24, 25]. The DTI measures direction and magnitude of water diffusion based on the data obtained from 6 or more gradient directions as opposed to 3 directions in DWI. The water movement within the white matter tracts is mainly restricted across the myelin sheaths, a principal contributor to directionally dependent water diffusion, that is, anisotropy. Mathematic indices such as fractional anisotropy (FA) derived from DTI data can imply microstructural integrity of brain tissue. Further application using fiber-tracking techniques can reveal the relationship between gliomas and adjacent white matter tracts (Figure 2), hence assisting surgical planning and monitoring tumor response to treatment [27–29]. However, measurements of FA for tumor grading may show conflicting results. Inoue et al. reported that the FA values of low-grade gliomas are significantly lower than those of high-grade by a threshold of 0.188 [30], while Goebell et al. showed low FA ratios in the tumor centers of both low-grade and high-grade gliomas [31]. In the peritumoral region, the T2-weighted hyperintense area surrounding the high-grade glioma, the FA value is typically reduced resulting from a combination of perifocal edema, tumor mass effect, and invasion of tumor cells [28, 32]. Low-grade gliomas tend to deviate, rather than destruct (Figure 3) or infiltrate (Figure 4), the adjacent white matter [28]. Therefore, FA value is less reduced in low-grade gliomas.

**Figure 5:**
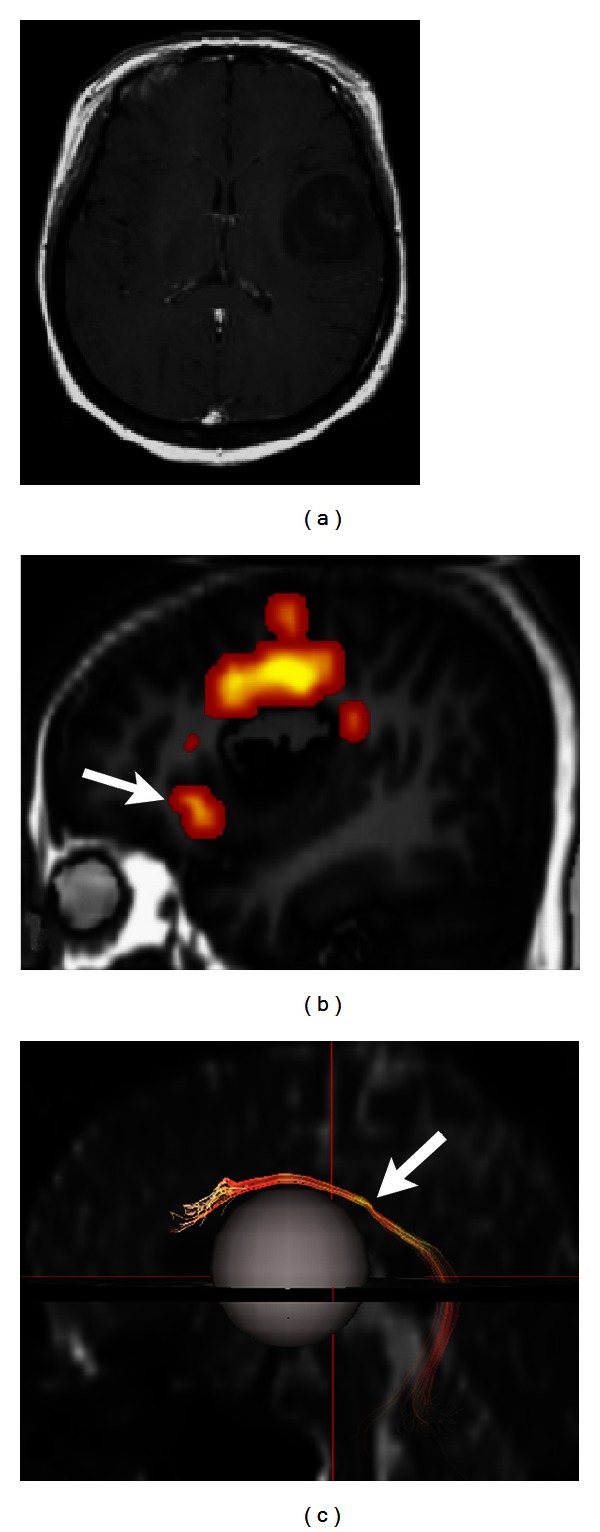
(a) Contrast-enhanced T1-weighted image shows an oligodendroglioma, WHO grade II, in the left frontal lobe. (b) Functional MR imaging and DTI tractography (c) demonstrate the activation of Broca's area (arrow in (b)) anterior to the tumor and the elevated arcuate fasciculus (arrow in (c)), respectively. The grey sphere in Figure 5(c) indicates the location of tumor.

Gliomas may affect both the functional cortex and the corresponding white matter tracts. The combination of the DTI and functional MR imaging can delineate an entire functional circuit (Figure 5), which can help surgical planning, reduce the surgery time, and minimize the need for intraoperative cortical stimulation [33]. However, the benefits remain to be proven in randomized trials.

### 2.3. Non-Gaussian Diffusion Kurtosis Imaging (DKI)

The computational algorithms of DTI are based on the ideal Gaussian distribution of water movement. However, this is not realistic in vivo as the brain represents a complex environment where the movement of water is restricted. In addition, the ADC values obtained from routine diffusion imaging using *b* value at 1000 sec/mm^2^ might only reflect extracellular water movement. Diffusion kurtosis imaging (DKI) is an extension of the DTI model capable of measuring the degree of non-Gaussian water diffusion [34]. The value of DKI has been shown in a study of Van Cauter et al. with kurtosis parameters contributing to better discrimination between high-grade and low-grade gliomas than with conventional diffusion parameters [35]. Further study is required to explore the role of DKI in evaluation of tumor invasiveness.

## 3. MR Imaging of Mitotic Activity

### 3.1. Gadolinium-Enhanced T1-Weighted Imaging

The mitotic activity or proliferation of gliomas significantly correlates with prognosis [36, 37]. Among various approaches available for assessing mitotic activity, MIB-1 antibody staining of the nuclear antigen Ki-67 is the most reliable and widely used method [38]. Ki-67 index has been shown to be a better prognostic indicator than histological grades [37, 39]. Several MR imaging techniques have been applied to correlate with tumoral mitotic activity. Among those techniques, conventional contrast-enhanced MR imaging was shown to be best correlated with Ki-67 index up to 8.1% in gliomas with contrast enhancement as opposed to 2.0% in those without enhancement [40]. However, the binary discrimination is insufficient in grading enhancing gliomas. As accelerated proliferative activity is coupled with high tumor cellularity and increased perfusion, DWI and perfusion MR imaging have been used to indirectly reflect mitotic activity of gliomas [24, 41, 42]. 

### 3.2. Tumor Activity and Image-Guided Biopsy by Proton MR Spectroscopy

**Figure 6:**
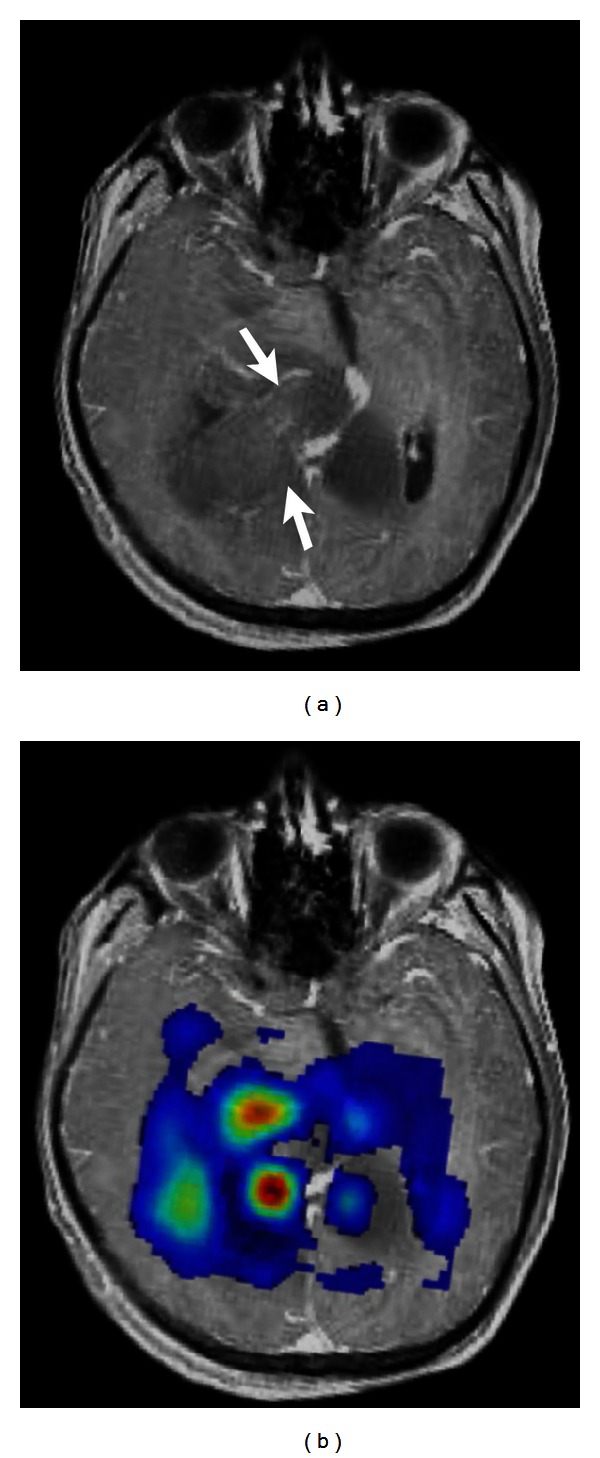
(a) Contrast-enhanced T1-weighted image depicts a glioblastoma involving the genu of corpus callosum. The arrows point two hot spots (targets) for stereotactic biopsy based on the regions of increased Cho/Cr ratios ((b) Cho/Cr map).

Proton MR spectroscopy (MRS) can noninvasively measure the brain metabolites in vivo. Some metabolites commonly used in clinical MRS study include but are not limited to N-acetyl aspartate (NAA, at 2.02 ppm), choline (Cho, at 3.2 ppm), creatine/phosphocreatine (Cr, 3.0 ppm), lactate (Lac, at 1.33 ppm), lipids (Lip, at 0.9–1.5 ppm range), and *myo*-inositol (mI, at 3.56 ppm). Although no tumor-specific metabolite has been labeled, the ratios of metabolites such as Cho/Cr ratio (Figure 6) have been used to assess cellular proliferation. Cho/Cr ratio has been shown to be parallel with the Ki-67 index in studies of single-voxel MRS [43–45]. In a study of multivoxel MRS, Tamiya et al. also showed a positive correlation between Cho/Cr ratio and Ki-67 index while NAA/Cho ratio has a negative relationship with the index [46]. 

**Figure 7:**
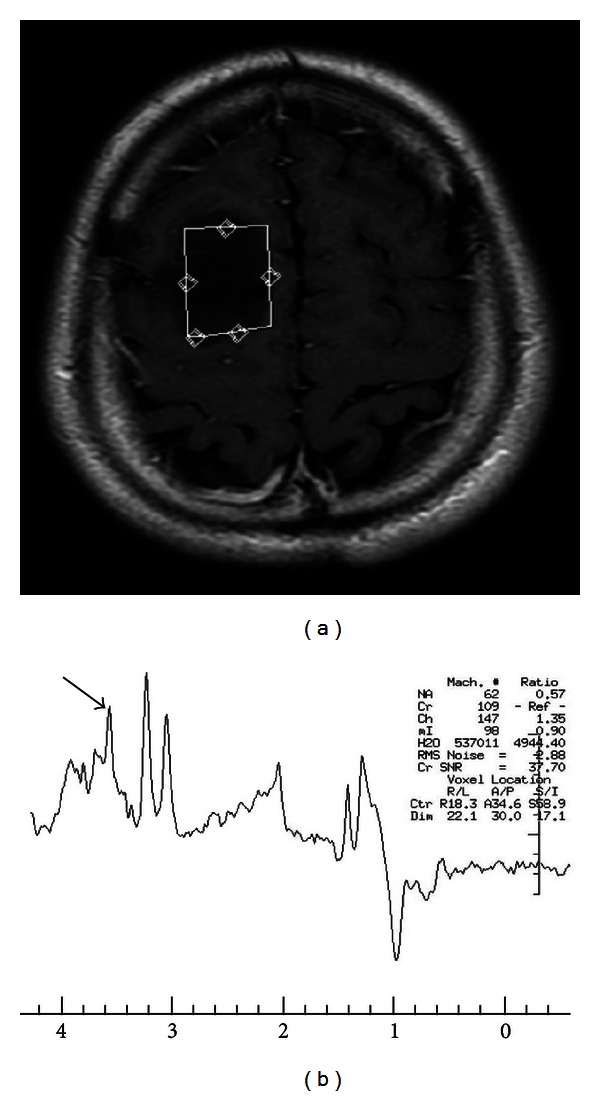
(a) Contrast-enhanced T1-weighted image shows a nonenhanced low-grade astrocytoma in the right superior frontal lobe with a high mI level on spectrum of proton MRS (arrow in (b)).

Another important clinical application of MRS is image-guided biopsy. Although conventional contrast-enhanced MR imaging is useful in delineating gliomas, the tumor regions where the most active mitotic activity exists may not always enhance and vice versa. Irrespective of contrast enhancement, chemical shift imaging using multivoxel ratios of Cho/NAA (the choline map) can be a valuable tool in locating high proliferative potential regions for accurate biopsy targets [47]. Furthermore, the resonances of Lac and Lip were found to be independent predictors of intermediate (Ki-67 index, 4−8%) and high (Ki-67 index, >8%) proliferative activities, respectively [48], while a higher level of mI is related to a lower grade of astrocytomas (Figure 7) [49].

## 4. Imaging of Angiogenesis

### 4.1. Perfusion-Weighted MR Imaging

Malignant gliomas are characterized by high degree of angiogenesis, a marker of histological grading system and one of the major therapeutic targets in the development of novel treatments [50]. The principal proangiogenic factor is vascular endothelial growth factor (VEGF), which can result in increased neovasculature, microvascular permeability, and vasodilatation [51–56]. The neovasculatures in gliomas function abnormally with irregularity of the endothelial lining and disruption of the blood-brain barrier (BBB) [57, 58]. The abnormal caliber and number of tumor vessels resulting from abnormal angiogenesis can be histologically measured by microvascular density or area (MVA), which may represent an independent prognostic biomarker [59]. However, the MVA calculation is time consuming and clinically arduous. A fast and noninvasive alternative in assessing MVA is dynamic susceptibility-weighted contrast-enhanced (DSC) MR imaging, which measures changes in tissue T2* following injection of contrast agent [60]. With a model that assumes that the contrast agent is restricted to the intravascular compartment, DSC MR imaging can generate a series of perfusion parameters, including relative cerebral blood volume (rCBV), referring to volume of blood in a given region of brain tissue, relative cerebral blood flow, referring to volume of blood per unit time passing through a given region of brain tissue, and mean transit time, referring to the average time for blood to pass through a given region of brain tissue. Among the parameters, rCBV is generally considered associated with tumor energy metabolism and provides a reliable estimate of tumor MVA [21, 61]. 

Dynamic contrast-enhanced (DCE) MR imaging is another perfusion method, which relies on the relaxivity effects, rather than the susceptibility effects assessed in DSC method, and measures T1 signal changes following injection of contrast agent. Because gadolinium exerts stronger relaxivity effects than the susceptibility ones, DCE method requires a smaller amount of contrast agent than DSC method does, allowing multiple repeated studies and better quantitation of the perfusion parameters [62, 63].

**Figure 8:**
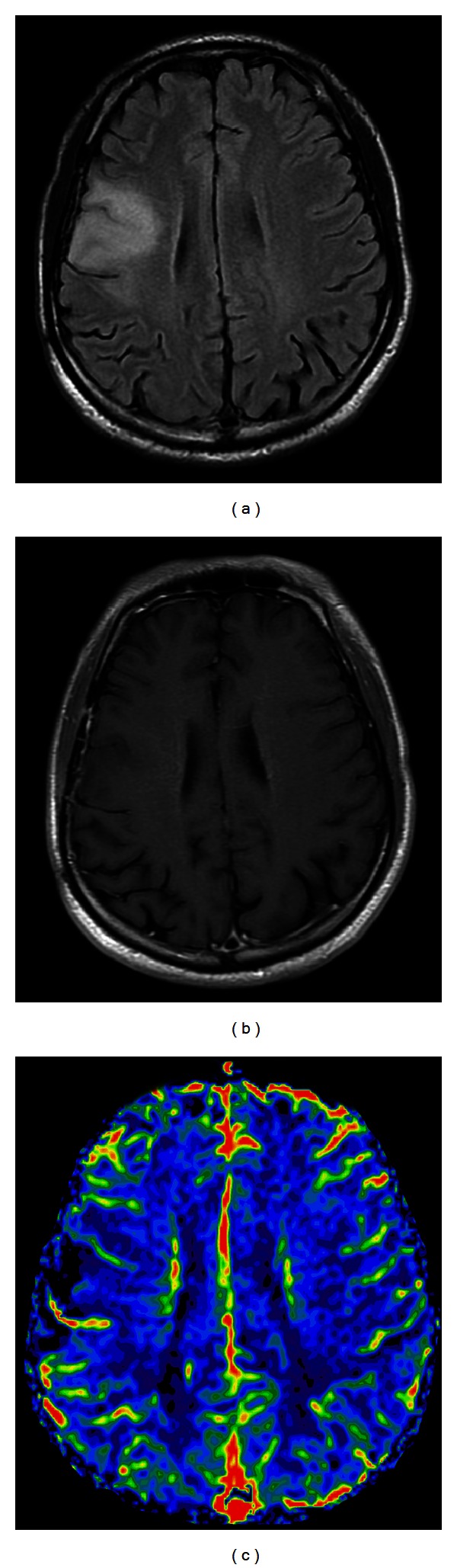
(a) FLAIR image shows a hyperintense low-grade glioma, WHO grade II, without significant contrast enhancement on T1-weighted image (b) or increase of the rCBV (c).

**Figure 9:**
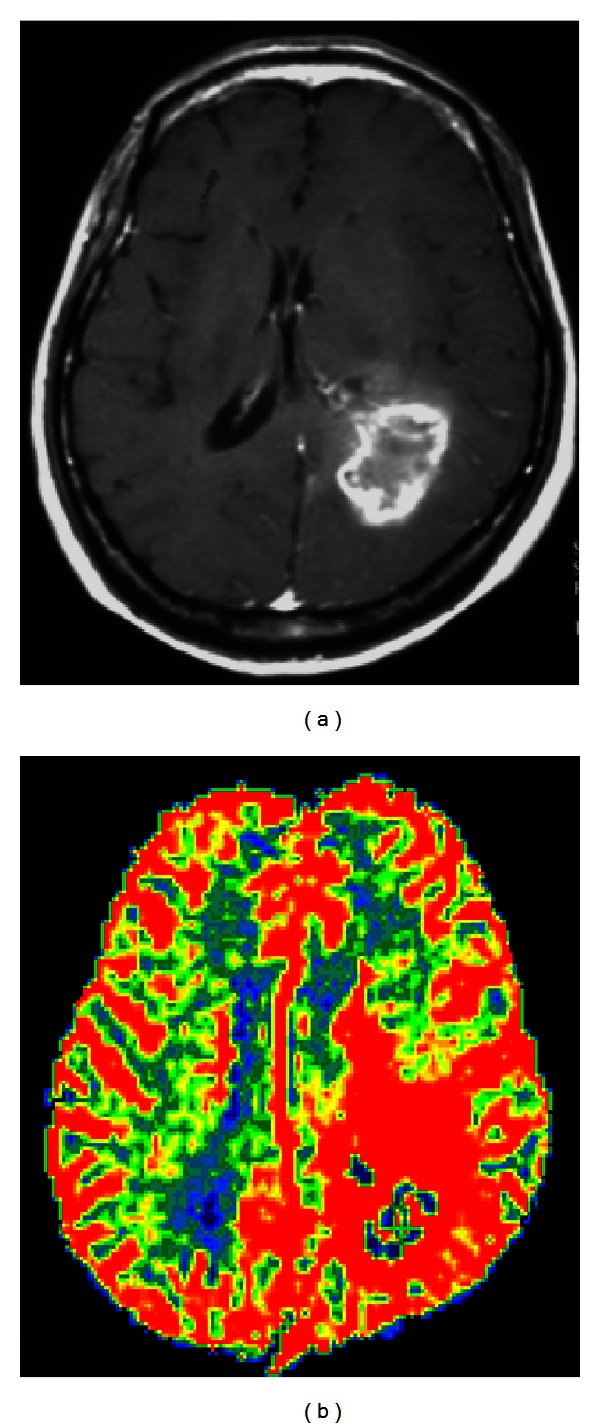
(a) Contrast-enhanced T1-weighted image demonstrates a ring-enhancing glioblastoma in the left parietal lobe with avid increase of rCBV (b) extending beyond the extent of the contrast enhancement.

**Figure 10:**
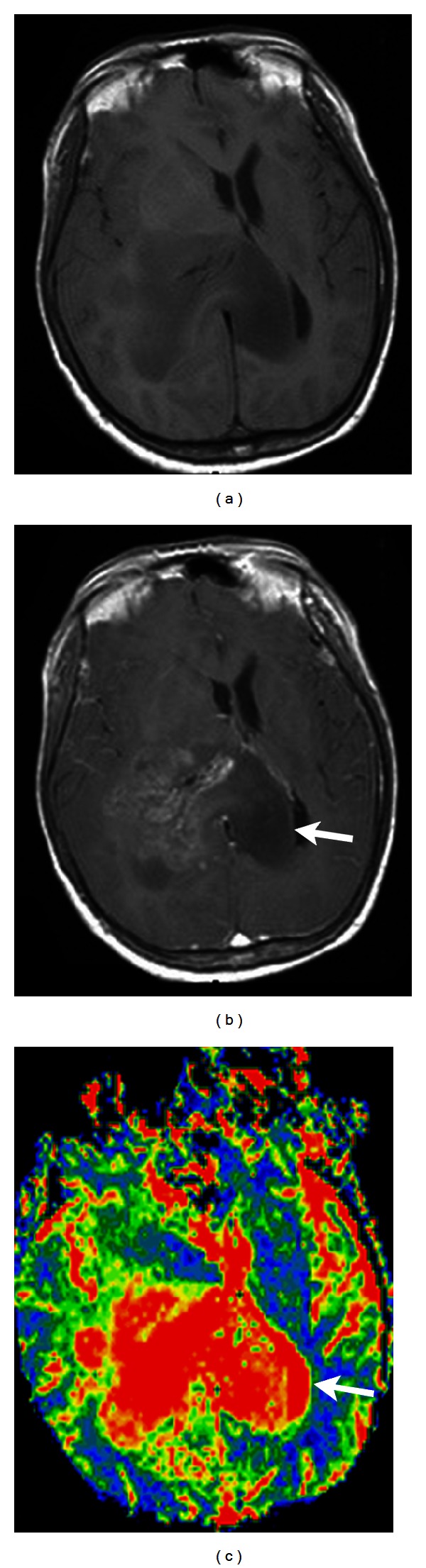
(a) Contrast-enhanced T1-weighted image shows a butterfly glioblastoma, involving the genu of corpus callosum. (b) The right aspect of the lesion appears to be heterogeneously contrast enhanced. (c) On rCBV map, the nonenhanced left aspect of the tumor (arrow) shows high rCBV (arrow in (c)). This helps differentiate tumor infiltration from perifocal edema.

Tumor neovasculatures tend to have leaky BBB, so the small molecular-weight gadolinium-based contrast agent readily extravasates, causing underestimation of the tumor rCBV. Consequently, the correlation between the rCBV and histologic tumor grading may not be always consistent unless rCBV is corrected for contrast extravasation [64]. Nonetheless, low-grade gliomas usually show no increase in tumor rCBV (Figure 8) while high-grade gliomas may demonstrate high rCBV that in some cases extends outside the contrast-enhancing portion of the tumor (Figure 9). Contrast enhancement in tumor may suggest impaired blood brain barrier with leakage of contrast agents into the extravascular spaces. Tumor with relatively intact BBB may show no enhancement. Therefore, conventional contrast-enhanced T1-weighted images and rCBV map can complement each other in outlining tumor extent and differentiating tumor from perifocal edema (Figure 10). 

As a standard of care, radiotherapy in combination of temozolomide chemotherapy for patients with newly diagnosed glioblastoma is related to enlarged enhancing areas on contrast-enhanced T1-weighted images without clinical worsening, a phenomenon known as pseudoprogression [65, 66]. Most often seen in patients with the concomitant radiochemotherapy, pseudoprogression can also occur in patients treated with radiotherapy or chemotherapy alone. In contrast to tumor progression, pseudoprogression is associated with a favorable prognosis [66–68]. Although follow-up conventional MR imaging studies can validate the initial worsening imaging findings, DSC MR imaging has been shown to be helpful in evaluating treatment effects in the first place. In a study of Sugahara et al. an enhanced lesion with a normalized rCBV ratio (tumor rCBV/contralateral tissue rCBV) higher than 2.6 suggests tumor recurrence while a normalized rCBV ratio lower than 0.6 implies pseudoprogression [69]. 

### 4.2. Capillary Permeability Imaging

**Figure 11:**
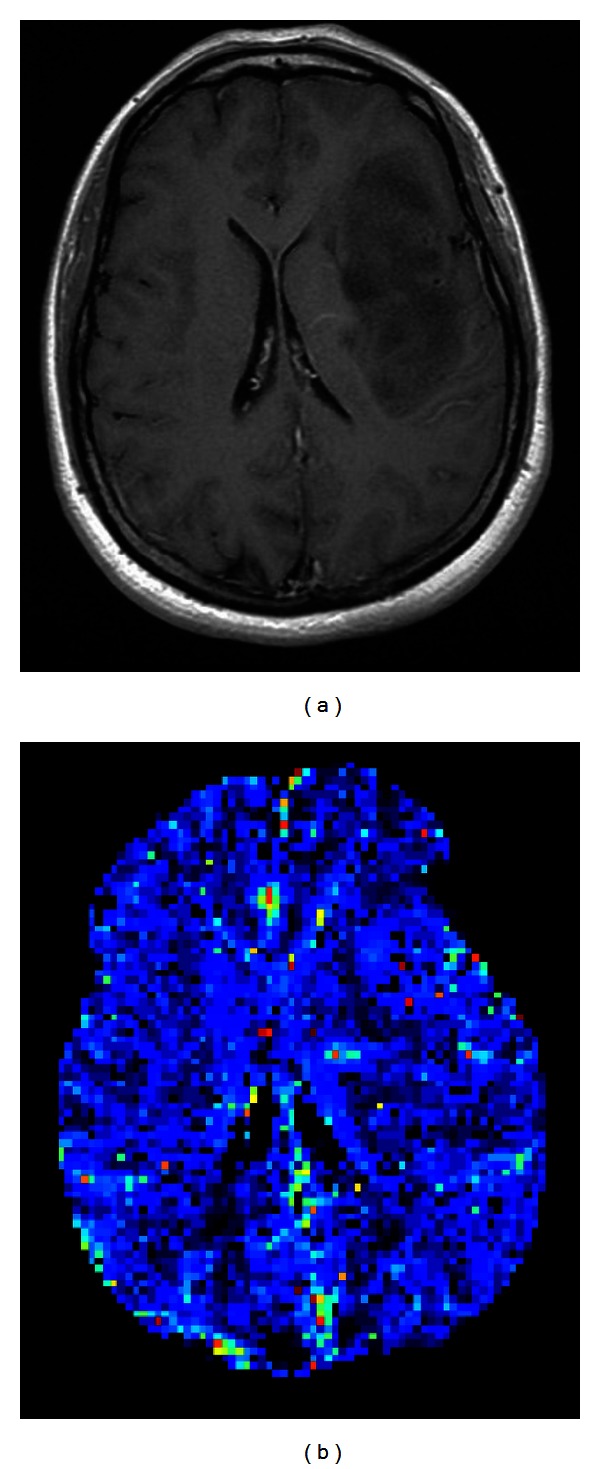
(a) Contrast-enhanced T1-weighted image depicts a nonenhanced astrocytoma, WHO grade II. (b) The *K* ^trans⁡^ map shows consistently no leakage of contrast medium in the tumor region, suggesting a low grade.

**Figure 12:**
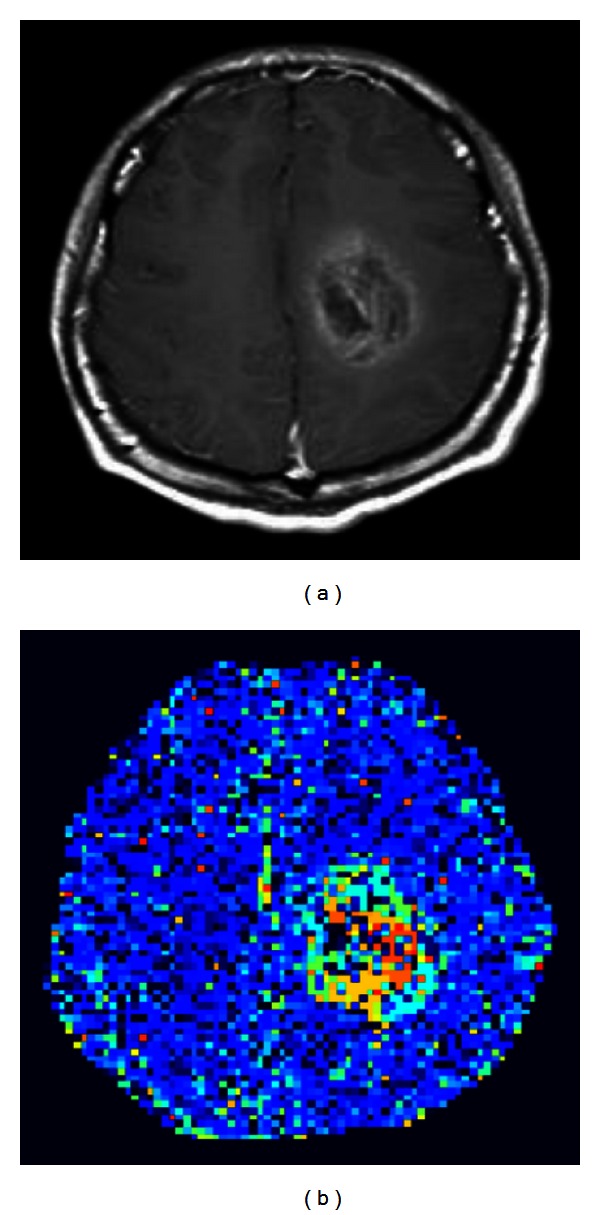
An anaplastic oligodendroglioma, WHO grade III, in the left frontal lobe shows contrast enhancement and leakage on T1-weighted image (a) and *K* ^trans⁡^ map (b), respectively.

**Figure 13:**
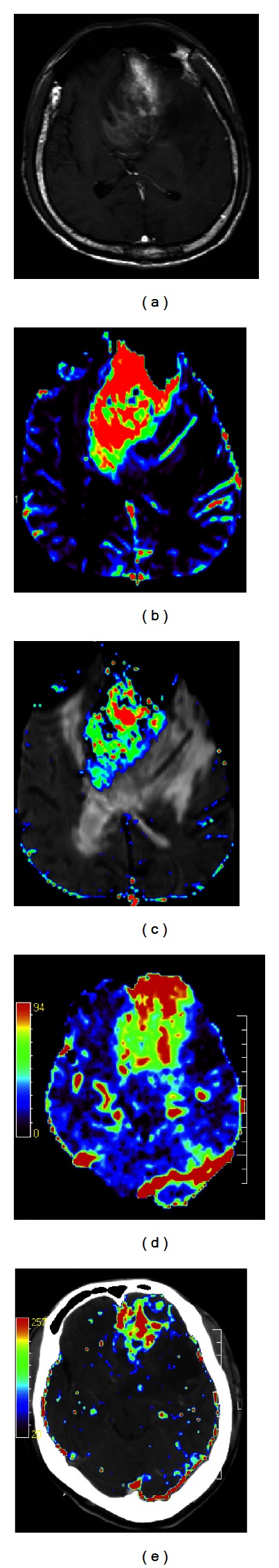
(a) Contrast-enhanced T1-weighted image shows an anaplastic oligodendroglioma, WHO grade III, involving the genu of corpus callosum and bilateral frontal lobes. rCBV (b) and *K* ^trans⁡^ (c) maps derived from MRI are comparable to those generated from CT perfusion imaging ((d) CT perfusion map; (e) CT *K* ^trans⁡^ map).

In addition to MVA, capillary permeability is another feature of angiogenesis in high-grade gliomas. MR imaging is capable of estimating the capillary permeability based on measuring the contrast leakage rate between the intravascular and extravascular spaces, known as the contrast transfer coefficient (*K* ^trans⁡^) [70, 71]. Although controversies remain among different models, the *K* ^trans⁡^ generally correlates with histological grading and length of survival in gliomas [72–74]. A typical low-grade glioma without increase of *K* ^trans⁡^ is shown in Figures 11 and 12 demonstrates high *K* ^trans⁡^ in a high-grade glioma. Although most researchers utilize MR imaging in assessing perfusion parameters of brain tumors, CT perfusion can be an alternative for patients contraindicated to MR imaging and provides parameters of tumor vascular physiology with various maps comparable to those generated by DSC MR perfusion imaging [75, 76].  Figure 13 shows comparable maps of rCBV and *K* ^trans⁡^ derived from CT and MR perfusion imaging.

### 4.3. Imaging of Tumor Response to Bevacizumab

Until recently, advances in molecular biology have shed light on the development of anti-VEGF monoclonal antibodies as a novel therapy for high-grade gliomas [77]. Bevacizumab is a FDA-approved monoclonal antibody that prevents the interaction of VEGF receptor tyrosine kinase and treats a variety of cancers, including glioblastomas [78–80]. However, in the maintenance of bevacizumab therapy, malignant gliomas inevitably recur and appear to be more aggressive with rebound edema [81]. The tumor response to bevacizumab treatment is unique in terms of imaging finding as the drug suppresses the enhancing component of tumor but not the non-enhancing and infiltrative tumor growth [82]. As a result, the traditional evaluation of treatment response, mostly defined by the McDonald criteria, based on contrast-enhanced CT or MR imaging, is not sufficient. New response criteria were developed for clinical trials of brain tumors by incorporating T2 and FLAIR changes on MR imaging to evaluate the unique infiltrative progression pattern of malignant gliomas [83]. 

### 4.4. Imaging of Microvasculature by Susceptibility-Weighted Imaging

Taking the advantage of high sensitivity to tumor microvasculature and hemorrhagic products, susceptibility-weighted imaging (SWI) is recently introduced to the array of imaging tools for evaluating angiogenesis. SWI is a high-resolution, three-dimensional, gradient-echo T2* MR technique that is blood oxygen level dependent and shows high sensitivity to paramagnetic substances, such as blood products, iron, and calcifications [84, 85]. In the study of Park et al., glioblastomas showed increased intratumoral susceptibility signals that are significantly different from low-grade gliomas and lymphomas with a specificity of 100% [86]. In an ultrahigh-field-strength (7T) gradient-echo MR study, serpentine hypointensities within gliomas (tumoral pseudoblush) concur with microvascular size and density in histopathological examination and were considered as a promising imaging biomarker for increased tumoral microvascularity [87]. Furthermore, SWI is also useful in the assessment of the microvascular change in patients undertaking bevacizumab therapy [88].

### 4.5. Molecular MR Imaging

With recent advances of nanotechnology and biotechnology, scientists are capable of binding paramagnetic transition metal ion chelates, mainly gadolinium chelates, or superparamagnetic iron oxide (SPIO) nanoparticles with biologically active targeting moieties and provide a new MR imaging tool to evaluate tumor-specific vasculatures in vivo [89]. SPIO nanoparticles are biodegradable iron oxide crystals with polymer coatings and have properties that cause microscopic filed inhomogeneity that dephase the neighboring proton magnetic moments and reduce the T2* relaxation time. In a study of Tomanek et al., an antibody-targeted MR contrast agent, consisting of SPIO and anti-insulin-like-growth-factor binding protein 7, was used to show abnormal vessels within a glioblastoma on T2-weighted images in a mouse model [90]. 

 As the key regulatory systems in angiogenesis of gliomas, VEGF and VEGF receptors are targeted mainly in radionuclide-based imaging and recently assessed by a molecular MR imaging probe, anti-VEGF receptor-2 monoclonal antibody conjugated with a gadolinium-based contrast agent, in a rat C6 glioma model by He et al. [91]. The expression of VEGF receptor-2 on vascular endothelial cells in glioma tissue was successfully visualized in vivo with the degree of the expression concurring that of the tumor blood volume [91]. 

## 5. MR Imaging of Tumor Necrosis

### 5.1. Contrast-Enhanced T1-Weighted Imaging and Proton MRS

**Figure 14:**
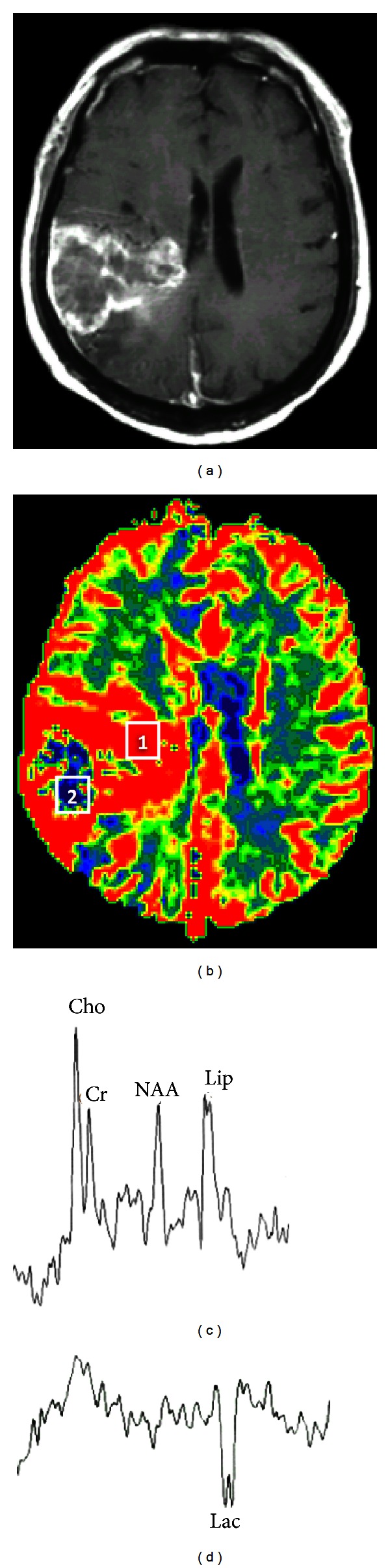
(a) Contrast-enhanced T1-weighted image demonstrates a necrotic glioblastoma in the right parietal lobe with increased rCBV (b) in the periphery of the tumor and peritumoral regions. (c) A single-voxel MRS, echo time 135 ms, obtained from region 1 of increased rCBV (b) shows a high Lip peak, suggesting micronecrosis. (d) MRS obtained from the region 2 of low rCBV (b) depicts an inverted Lac peak, representing hypoxia in the necrotic region.

Necrosis is the hallmark of glioblastoma and is caused by tumor hypoxia as a result of increased cell proliferation and mitotic activity, as well as insufficient tissue perfusion. On conventional contrast-enhanced T1-weighted images, tumor necrosis can be easily diagnosed with the fact that necrotic zones are typically less enhanced, giving the tumor an appearance of irregular rim enhancing mass (Figure 4(a)). However, imaging diagnosis of necrosis can be problematic in early stages or in micronecrosis in which the necrotic region may show to be enhanced or not enhanced at all. MRS is an imaging tool of choice to show characteristic metabolites accumulated in the necrotic regions, even when necrosis is not overtly seen on contrast-enhanced T1-weighted images. The anaerobic glycolysis and cell death with membrane breakdown in the hypoxic tumor can be revealed by the increased Lac and Lip peaks on MRS (Figure 14) [92–94]. The presence of Lip and/or Lac in high-grade gliomas has been found in a number of studies [95–98].

### 5.2. Radiation-Induced Necrosis and Tumor Recurrence

The differentiation between radiation necrosis and recurrent high-grade gliomas remains challenging despite advances of imaging modalities because both entities share similar imaging features, such as irregular rim-like contrast enhancement, mass effect, and vasogenic edema. Although guidelines based on experiences on conventional MR imaging were intended to resolve the dilemma [99, 100], advanced imaging techniques have been shown to provide more reliable and accessible differentiation between the two conditions. In an MRS study of Nakajima et al., the Lac/Cho ratios are significantly higher in radiation necrosis (2.35 ± 1.81 (mean ± standard deviation)) than those in tumor recurrence (0.63 ± 0.25) [101]. DSC perfusion MR imaging was also shown to have higher relative peak height and rCBV in patients with recurrent glioma than in patients with radiation necrosis [102]. In a study of Larsen et al., a threshold of 2.0 mL/100 g for CBV was suggested to have 100% sensitivity and specificity for detecting gliomas in progression [103].

 Another clinical dilemma is the differentiation between ring-enhancing brain abscess and tumor necrosis on T1-weighted images. DWI is routinely used to differentiate the two conditions by showing restricted water diffusion of the high viscosity and cellularity of pus cells in the abscess cavity. However, the restricted diffusion within ring-enhancing lesions is not pathognomonic for brain abscess, and a small number of glioblastomas may show restricted diffusion in the necrotic regions, probably resulting from a various combination of intratumoral hemorrhage, cytotoxic edema, or superimposed pyogenic infection [104, 105]. The dilemma has recently been successfully resolved by the application of perfusion MR imaging, which reveals distinct pathophysiological alterations between brain abscess and glioblastoma. In a prospective study Chiang et al. showed decreased rCBV in the necrotic wall of the abscess where regional poor vascularity exists and increased rCBV in the periphery of the high-grade gliomas owing to the presence of active angiogenesis [106]. Furthermore, a characteristic dual rim sign, presumably resulting from the granulation tissue, on SWI, found only along the wall of brain abscess but not in glioblastomas, has been proposed by Toh et al. to effectively differentiate the two conditions [107].

## 6. Conclusion

Advanced MR imaging techniques can potentially help evaluate the underlying key histopathological features of gliomas by showing the physiologic changes and metabolic activities, thus improving diagnosis and tumor grading. These functional tools help in better understanding of the tumor behavior and also provide a new window to guide and monitor the treatment of gliomas. Application of these imaging techniques could lead to sophisticated and personalized patient care.
